# Prognostic significance of enlarged paraaortic lymph nodes detected during left-sided colorectal cancer surgery: a single-center retrospective cohort study

**DOI:** 10.1186/s12957-020-02118-w

**Published:** 2021-01-12

**Authors:** Jaram Lee, Hyeong-min Park, Soo Young Lee, Chang Hyun Kim, Hyeong Rok Kim

**Affiliations:** Department of Surgery, Chonnam National University Hwasun Hospital and Medical School, 322 Seoyang-ro Hwasun-eup, Hwasun-gun, Jeonnam 58128 South Korea

**Keywords:** Colorectal cancer, Paraaortic lymph node metastasis, Paraaortic lymph node dissection

## Abstract

**Background:**

Very few studies have been conducted on the treatment strategy for enlarged paraaortic lymph nodes (PALNs) incidentally detected during surgery. The purpose of this study was to investigate the benefit of lymph node dissection in patients with incidentally detected enlarged PALNs.

**Methods:**

We retrospectively reviewed patients with left colon and rectal cancer who underwent surgical resection with PALN dissection between January 2010 and December 2018. The predictive factors for pathologic PALN metastasis (PALNM) were analyzed, and survival analyses were conducted to identify prognostic factors.

**Results:**

Among 263 patients included, 19 (7.2%) showed pathologic PALNM and 5 (26.33%) had enlarged PALNs incidentally detected during surgery. These 5 patients accounted for 2.2% of 227 patients who had no evidence of PALNM on preoperative radiologic examination. Radiologic PALNM (odds ratio [OR] 12.737, 95% confidence interval [CI] 3.472–46.723) and radiologic distant metastasis other than PALNM (OR = 4.090, 95% CI 1.011–16.539) were independent predictive factors for pathologic PALNM. Pathologic T4 stage (hazard ratio [HR] 2.196, 95% CI 1.063–4.538) and R2 resection (HR 4.643, 95% CI 2.046–10.534) were independent prognostic factors for overall survival (OS). In patients undergoing R0 resection, pathologic PALNM was not associated with 5-year OS (90% vs. 82.2%, *p* = 0.896).

**Conclusion:**

Dissection of enlarged PALNs incidentally detected during colorectal surgery may benefit patients with favorable survival outcomes.

## Introduction

Colorectal cancer is the third most common cancer in men and the second most common cancer in women, with 1.8 million new cases in 2018 according to the World Health Organization GLOBOCAN database [1]. Approximately 22% of patients with colorectal cancer are diagnosed at stage IV. The 5-year relative survival of colorectal cancer patients of all stages is approximately 67%, whereas the 5-year relative survival of stage IV patients is only 15% [2].

According to the American Joint Committee on Cancer (AJCC) classification, paraaortic lymph node (PALN) metastasis (PALNM) is categorized as distant metastasis (M1), and therefore stage IV [3]. PALNM occurs in approximately 2% of colorectal cancer patients [4]. Previous studies have shown that patients with PALNM have a significantly lower survival rate than those without PALNM [5–8]. Although several studies have shown that PALN dissection (PALND) improves survival in patients with PALNM, there has been no clearly defined standard treatment for PALNM. Moreover, very few studies have been conducted on the treatment strategy for enlarged PALNs incidentally detected during colorectal cancer surgery.

Therefore, we designed this study to investigate the probability of pathologic PALNM and the benefit of PALND in patients with enlarged PALNs incidentally detected during colorectal cancer surgery.

## Methods

We retrospectively reviewed patients with primary colorectal cancer who underwent surgical resection at our institution between January 2010 and December 2018. This study was approved by the institutional review board of our institution (CNUHH-2020-157).

Patients with left-sided colon and rectal cancer who underwent colorectal surgery with PALND were included in this study. We included all patients with retrieved PALNs regardless of whether or not PALNM was suspected on preoperative radiologic examination. Patients without suspected PALNM in the preoperative imaging study were classified into the incidentally detected PALN group.

The clinicopathologic factors of the included patients were retrospectively collected through a review of medical records, which included demographic data, preoperative evaluation, operative findings, pathologic characteristics, and postoperative outcomes. Preoperative evaluation included serum levels of carcinoembryonic antigen (CEA), colonoscopy, abdominopelvic computed tomography (CT), and rectal magnetic resonance imaging. Positron emission tomography (PET) was selectively performed when distant metastasis was suspected. PALNM was suspected when the PALN was > 8 mm or > 5 mm and showed heterogeneity and an irregular outer border on abdominopelvic CT scan. In PET-CT, the presence of PALNs with higher fluorodeoxyglucose uptake than adjacent normal organs was considered to indicate PALNM.

All patients underwent mechanical bowel preparation before surgery. Second-generation cephalosporin was routinely administered within 1 h before surgery as an empirical antibiotics. In most cases, D3 dissection with high ligation of the inferior mesenteric artery was performed. For tumors below the peritoneal reflection, total mesorectal excision was performed. The detailed surgical technique has been previously described [9].

The extent of PALND was determined at the surgeon’s discretion. In cases of highly suspicious PALNM, the PALNs were dissected from the aortic bifurcation to the lower border of the renal vein. In some cases with a single suspicious metastatic PALN, only the suspicious PALN was dissected. In most cases, the surgical procedure including PALND was performed using a laparoscopic approach.

R0 resection was defined as no macroscopic or microscopic residual tumor after surgery, and with no remnant tumor observed on follow-up radiologic examination. R2 resection was defined as a gross residual tumor remaining after surgery or a remnant tumor observed on the first follow-up CT. Histopathologic examination was performed by a specialized pathologist according to standard procedures. The classification system of the AJCC (seventh edition) was used to determine the pathologic stage. Adjuvant chemotherapy was recommended for advanced colorectal cancer, after considering the patient’s general condition. Follow-up examinations included chest CT, abdominopelvic CT, and serum CEA test every 6 months and colonoscopy every 2 years. Recurrence was defined as recurrent disease on clinical, radiologic, or pathologic examination. Overall survival (OS) was defined as the period from surgery to death from any cause.

Categorical variables were compared using the *χ*^2^ test or Fisher’s exact test, and continuous variables were compared using Student’s *t* test. Logistic regression analysis was used to investigate the predictive factors for PALNM. Survival outcomes were compared using the Kaplan–Meier method and log-rank test. Multivariate analysis with a Cox proportional hazard model was used to identify prognostic factors for OS. Variables that were significant at *p* < 0.10 in the univariate analysis were entered into a multivariate model. Statistical significance was set at *p* < 0.05. All statistical analyses were performed using IBM SPSS version 23 (IBM, Armonk, NY).

## Results

**Fig. 1:**
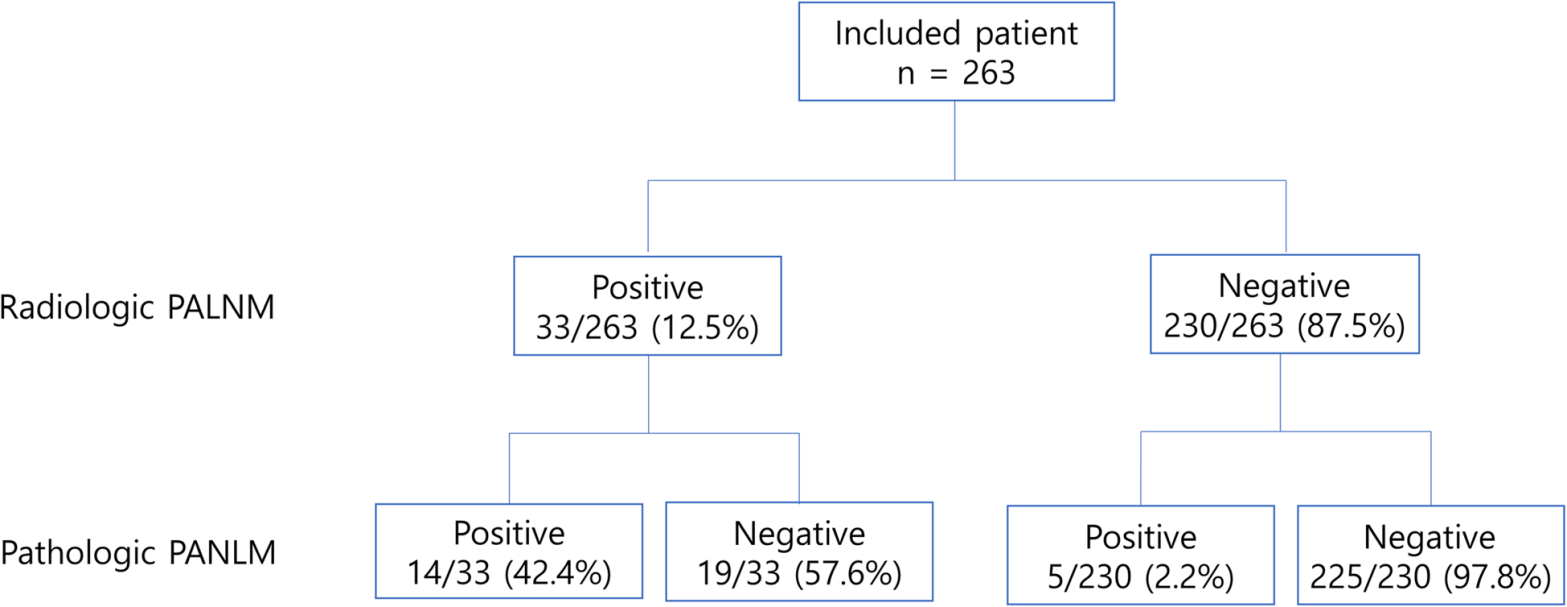
Study flow chart (PALNM, paraaortic lymph node metastasis)

A total of 263 patients with left colon and rectal cancer who underwent PALND were included, and 19 (7.2%) of them showed pathologic PALNM. Of these 19 patients, 14 (73.7%) were suspected to have PALNM on preoperative radiologic examination, and 5 (26.3%) had enlarged PALNs incidentally detected during surgery. These 5 patients accounted for 2.2% of the 227 patients who had no evidence of PALNM on preoperative radiologic examination (Fig. 1). Frozen-section biopsy for PALNs was performed in 29 (11.0%) of the 263 included patients. Of the 29 patients, 5 (17.2%) had metastatic adenocarcinoma in frozen-section analysis, 4 of whom had suspicious PALNM on preoperative imaging study.

Table 1 shows the clinicopathological characteristics according to pathologic PALNM. Regarding preoperative characteristics, higher clinical T stage (cT4 29.0% vs. cT1 0.0%, cT2 3.2%, cT3 3.8%; *p* < 0.001), higher clinical N stage (cN2 17.6%, vs. cN0 2.1%, cN1 1.3%; *p* < 0.001), radiologic PALNM (42.4% vs. 2.2%, *p* < 0.001), and radiologic distant metastasis other than PALNM (31.8% vs. 5.0%, *p* < 0.001) were associated with a higher incidence of pathologic PALNM. Regarding pathologic characteristics, larger tumor size (> 5 cm), lymphovascular invasion and poor differentiation were associated with pathologic PALNM.

**Table 1:** Characteristics according to pathologic PALNM

Characteristics		Pathologic PALNM (−) (*n* = 244)	Pathologic PALNM (+) (*n* = 19)	*p*
Preoperative				
Sex	Male	158 (94.0%)	10 (6.0%)	0.325
	Female	86 (90.5%)	9 (9.5%)	
Age (years)		63.61 ± 11.846	63.37 ± 10.761	0.931
BMI (kg/m^2^)		22.2 ± 2.7	22.2 ± 2.6	0.929
ASA	1, 2	224 (92.9%)	17 (7.1%)	0.665
	3, 4	20 (90.9%)	2 (9.1%)	
Preoperative	< 5	160 (94.7%)	9 (5.3%)	0.133
CEA (ng/mL)	≥ 5	82 (89.1%)	10 (10.9%)	
Clinical T stage	cT1	13 (100%)	0 (0.0%)	< 0.001
	cT2	30 (96.8%)	1 (3.2%)	
	cT3	179 (95.2%)	9 (4.8%)	
	cT4	22 (71.0%)	9 (29.0%)	
Clinical N stage	cN0	94 (97.9%)	2 (2.1%)	< 0.001
	cN1	75 (98.7%)	1 (1.3%)	
	cN2	75 (82.4%)	16 (17.6%)	
Radiologic	Negative	225 (97.8%)	5 (2.2%)	< 0.001
PALNM	Positive	19 (57.6%)	14 (42.4%)	
Radiologic DM	Negative	229 (95.0%)	12 (5.0%)	< 0.001
Other than PALNM	Positive	15 (68.2%)	7 (31.8%)	
Tumor location	Colon	107 (93.0%)	8 (7.0%)	0.882
	Rectum	137 (92.6%)	11 (7.4%)	
Operative				
Surgical procedure	Open	6 (60.0%)	4 (40.0%)	0.003
	Laparoscopy	238 (94.1%)	15 (5.9%)	
Operative time (min)	< 180	197 (93.8%)	13 (6.2%)	0.232
	≥ 180	47 (88.7%)	6 (11.3%)	
Pathologic				
Tumor size (cm)	< 5	130 (96.3%)	5 (3.7%)	0.03
	≥ 5	113 (89.0%)	14 (11.0%)	
Lymphovascular	Negative	203 (97.6%)	5 (2.4%)	< 0.001
Invasion	Positive	41 (74.5%)	14 (25.5%)	
Perineural	Negative	148 (94.9%)	8 (5.1%)	0.146
Invasion	Positive	96 (89.7%)	11 (10.3%)	
Differentiation	w/d, m/d	227 (93.4%)	16 (6.6%)	0.031
	p/d	7 (70.0%)	3 (30.0%)	

Data are presented as mean ± standard deviation or number (percentage)

*PALNM* Paraaortic lymph node metastasis; *BMI* Body mass index; *ASA* American Society of Anesthesiologists; *CEA* Carcinoembryonic antigen; *DM* Distant metastasis; *w/d* Well differentiated; *m/d* Moderately differentiated; *p/d* Poorly differentiated

In multivariate analysis, radiologic PALNM (odds ratio [OR] 12.737, 95% confidence interval [CI] 3.472–46.723, *p* < 0.001) and radiologic distant metastasis other than PALNM (OR 4.090; 95% CI, 1.011–16.539; *p* = 0.048) were independent predictive factors for pathologic PALNM. However, higher clinical T stage and N stage were not predictive factors for pathologic PALNM in the multivariate analysis (Table 2).

**Table 2:** Multivariate analysis of predictive factors for pathologic PALNM

Variables	Pathologic PALNM	
	OR (95% CI)	*p*
Clinical T stage (cT4 vs. cT1–3)	3.182 (0.865–11.701)	0.081
Clinical N stage (cN2 vs. cN0–1)	2.705 (0.577–12.671)	0.207
Radiologic PALNM (positive vs. negative)	12.737 (3.472–46.723)	< 0.001
Radiologic DM other than PALNM (positive vs. negative)	4.090 (1.011–16.539)	0.048

*PALNM* Paraaortic lymph node metastasis; *OR* Odds ratio; *CI* Confidence interval; *DM* Distant metastasis

Table 3 shows postoperative complications. The overall 30-day morbidity rate was 18.2% (48/263), and the most common complication was prolonged postoperative ileus (6.8%). Anastomotic leakage occurred in 4.2% (11/263) of all the included patients. No postoperative mortality was recorded.

**Table 3:** Postoperative complications

Morbidity	*n* (%)
Total	48 (18.2%)
Bleeding	2 (0.8%)
Ileus	18 (6.8%)
Anastomotic leakage	11 (4.2%)
Urinary dysfunction	13 (4.9%)
Pneumonia	1 (0.4%)
Wound infection	6 (2.3%)
Deep SSI	5 (1.9%)

*SSI* Surgical site infection

**Fig. 2:**
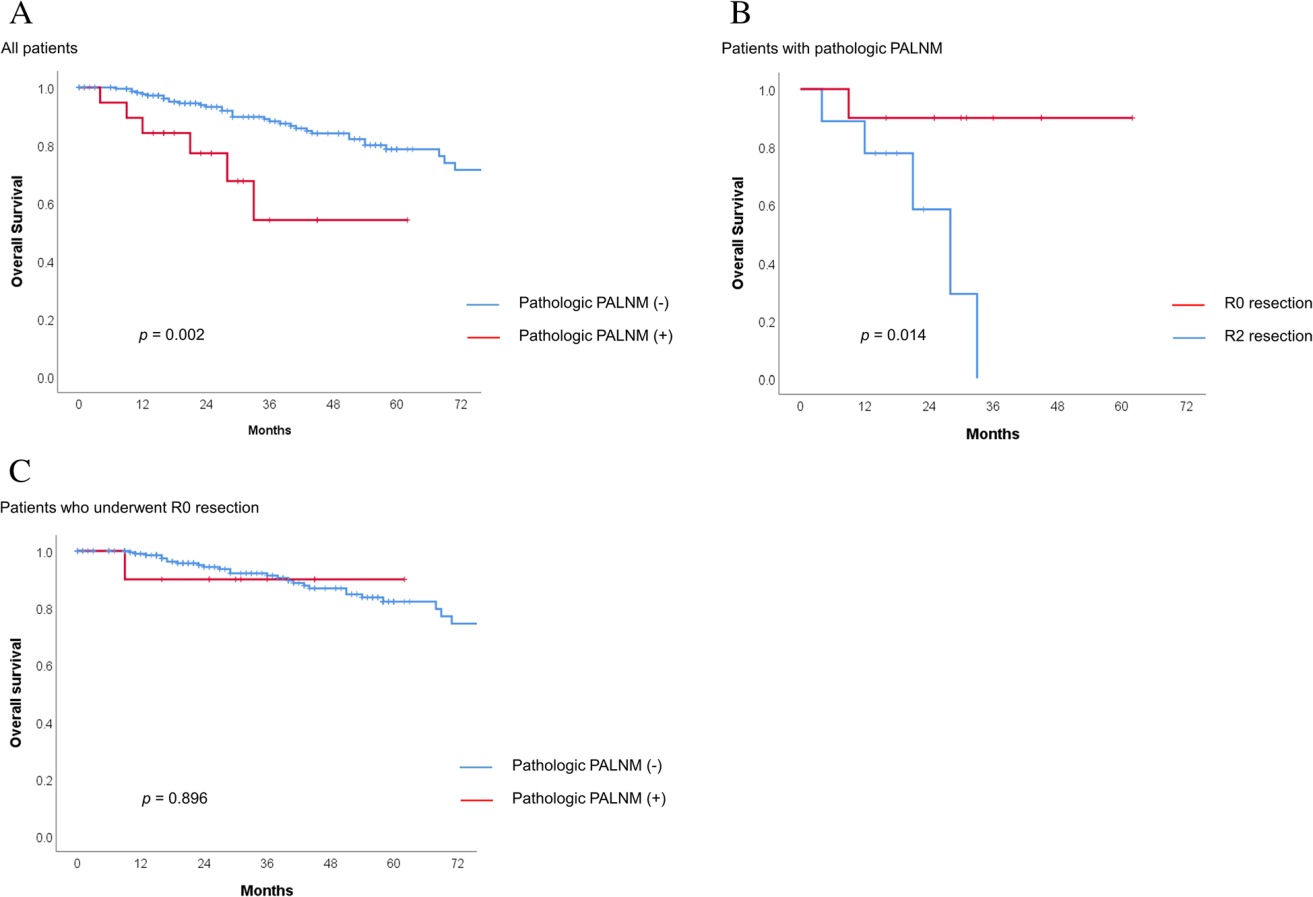
Overall survival for (**a**) all patients according to pathologic paraaortic lymph node metastasis (PALNM); (**b**) patients with pathologic PALNM according to radicality of resection; (**c**) patients who underwent R0 resection according to pathologic PALNM

Prognostic factors for OS are described in Table 4. In univariate survival analysis, pathologic T4 stage (37.3% vs. 82.1%, *p* < 0.001), pathologic N1-2 stage (65.8% vs. 87.7%, *p* = 0.001), M1 stage (38.8% vs.81.5%, *p* < 0.001), pathologic PALNM (54.0% vs. 78.6%, *p* = 0.002), and R2 resection (12.8% vs. 82.3%, *p* < 0.001) were associated with poorer OS (Table 4, Fig. 2a). Because M stage and R0 resection were highly correlated with each other (correlation coefficient = 0.727, *p* < 0.001), M stage was excluded from the multivariable survival analysis. In the multivariable analysis, pathologic T4 stage (hazard ratio [HR] 2.196, 95% CI 1.063–4.538, *p* = 0.034) and R0 resection (HR 4.643, 95% CI 2.046–10.534, *p* < 0.001) were independent prognostic factors for OS.

**Table 4:** Prognostic factors for overall survival by univariate and multivariate analyses

Variables	*n*	Univariate analysis		Multivariate analysis	
		5-year OS (%)	*p*	HR (95% CI)	*p*
Pathologic T stage					
T4	49	37.6	< 0.001	2.196 (1.063–4.538)	0.034
T1–3	214	82.1			
Pathologic N stage					
N1-2	137	65.8	0.001	2.064 (0.961–4.434)	0.063
N0	126	87.7			
M stage					
M1	32	38.8	< 0.001		
M0	231	81.5			
pathologic palnm					
Positive	19	54.0	0.002	1.367 (0.505–3.703)	0.538
Negative	244	78.6			
R0 resection					
R2	20	12.8	< 0.001	4.643 (2.046–10.534)	< 0.001
R0	243	82.3			

*OS* Overall survival; *HR* hazard ratio; *CI* Confidence interval; *PALNM* Paraaortic lymph node metastasis

Among all included patients, 20 (7.6%) underwent R2 resection. Of 19 patients with pathologic PALNM, 9 patients (47.4%) underwent R0 resection and 10 patients (52.6%) underwent R2 resection. When comparing the survival outcome according to the radicality of resection in patients with PALNM, OS was significantly higher in patients undergoing R0 resection (5-year OS, 90.0% vs. 0.0%; *p* = 0.014) (Fig. 2b). In the subgroup analysis of patients who underwent R0 resection, no difference in 5-year OS (90.0% vs. 82.2%, *p* = 0.896) was found between patients with and without pathologic PALNM (Fig. 2c).

## Discussion

In the present study, we investigated the necessity of PALND in patients with incidentally detected enlarged PALNs. The results showed that even in patients with no evidence of PALNM on preoperative radiologic examination, pathologic PALNM was identified in a small but non-negligible proportion (2.2%). Patients with pathologic PALNM showed poorer OS than those without pathologic PALNM. However, when R0 resection was achieved, patients with pathologic PALNM had comparable OS to those without pathologic PALNM.

PALNM is a known poor prognostic factor in patients with colorectal cancer [4–7]. In cases of liver or lung metastasis, radical resection of the metastatic lesion is known to be beneficial for survival [10–12]. However, there is no established standard treatment for PALNM, which is relatively rarer than liver or lung metastasis [7, 8, 13]. Recently, several studies have reported that PALND increased survival in patients with PALNM [6, 7, 13–15]. For example, Choi et al. reported that the 5-year OS of the PALND group was significantly higher than that of the control group (53.4% vs. 12%, *p* = 0.045) [6]. Ogura et al. reported that the 5-year cancer-specific survival was significantly higher in the R0 resection group than in the control group with palliative resection in patients with extra-regional lymph node metastasis [16]. Gagniere et al. reported the outcome of radical retroperitoneal lymphadenectomy in patients with retroperitoneal nodal metastases from colorectal cancer [17]. They suggested that the 5-year OS of patients who underwent lymphadenectomy was significantly higher than that of patients treated with non-surgical treatment. However, some authors have reported that PALND is not recommended because of its technical difficulties and lack of survival benefit [18]. Others reported a high recurrence rate after PALND even when performed in selective patients with PALNM [19].

Determining the presence of PALNM through a preoperative imaging study before surgery and establishing an appropriate surgical plan are essential. In this study, radiologic PALNM was identified as a predictive factor for pathologic PALNM in the multivariable analysis. Nakai et al. evaluated the diagnostic value of CT and PET/CT in predicting PALNM [20]. When the diagnosis was based on CT combined with PET/CT, the diagnostic ability of PET/CT was 93.8% in patients who had no predictive CT findings. However, in patients with a suspected lesion on CT, the diagnostic ability of PET/CT was decreased to 70.6%. According to a study by Wong et al., among 264 patients suspected of having PALNM on radiologic examination, 118 patients showed positive PALNM in pathology, and the positive predictive value was only 44.7% [5]. Similarly, in the present study, the accuracy of preoperative imaging in predicting PALNM was 89.7%, whereas the positive predictive value was only 42.4%. Therefore, it is not easy to accurately diagnose PALNM based on preoperative imaging findings.

Surgeons often incidentally detect enlarged PALNs during surgery and deliberate about whether PALND should be performed. However, no standard treatment has been established for the management of incidentally detected enlarged PALNs in colorectal cancer patients. Importantly, in the present study, there were five (2.2%) false-negative cases in which PALNM was not detected on preoperative imaging but PALND was performed incidentally. In addition, a significant difference in OS was observed between the R0 and R2 resection groups (90.0% vs. 0.0%, *p* = 0.014). When R0 resection was achieved in patients with PALNM their OS was comparable to that of patients without PALNM (90.0% vs. 82.2%, *p* = 0.896). Therefore, we recommend performing PALND not only in patients with radiologically suspected PALNM but also in patients with enlarged PALNs incidentally detected during surgery, especially in patients with radiologic distant metastasis other than PALNM.

Frozen-section biopsy of an enlarged PALN may be helpful to decide whether to proceed with further PALND. In the present study, frozen-section biopsy was performed in only 11.0% (29/263) of patients, and 5 (17.2%) of them showed PALNM. Because the present study was retrospective, it was impossible to investigate whether the extent of surgery changed according to the result of frozen-section biopsy. However, given the fact that PALNM had not been found in 24 (83.8%) of the 29 patients with frozen-section biopsy, frozen-section biopsy may be helpful to avoid unnecessary wider PALND, preventing possible long-term complications.

Several studies have reported that retroperitoneal lymph node dissection is related to sexual dysfunction due to hypogastric nerve injury, chylous ascites, and lymphoceles [21, 22]. According to a systematic review by Wong et al. [5], the postoperative complication rate of PALND ranged from 7.8 to 33%. We observed a postoperative complication rate of 18.2%, with no postoperative mortality. The morbidity rate in the present study was comparable to that in our previous studies (13.9–31.2%) on rectal cancer surgery [23, 24]. PALND can be safely performed with minimal additional surgical complications.

This study had several limitations. First, this study was designed as a retrospective review of data from a single institution. Because PALND was decided and performed by four surgeons, the indication for PALND was not standardized, which might have led to a selection bias. Second, as the number of patients with pathologic PALNM was small, the results of the multivariate analysis need to be interpreted with caution. Third, we could not evaluate late complications such as ejaculatory dysfunction and reduced quality of life. Despite these limitations, the present study has a strength because very few studies have reported on the probability of pathologic PALNM in patients with incidentally detected PALNs.

## Conclusion

The incidence of pathologic PALNM in patients with enlarged PALNs incidentally detected during colorectal cancer surgery was not negligible (2.2%). If R0 resection can be achieved, patients with PALNM can show a relatively good prognosis. Therefore, we recommend performing PALND when an enlarged PALN is incidentally detected during surgery for the purpose of R0 resection.

## Data Availability

The data that support the findings of this study are available from the corresponding author, upon reasonable request.

## Abbreviations

AJCC: American joint committee on cancer
CEA: Carcinoembryonic antigen
CT: Computed tomography
HR: Hazard ratio
OR: Odds ratio
OS: Overall survival
PALNs: Paraaortic lymph nodes
PALNM: Paraaortic lymph node metastasis
PALND: Paraaortic lymph node dissection
PET: Positron emission tomography
